# Reversal of Propofol-induced Depression of the Hypoxic Ventilatory Response by BK-channel Blocker ENA-001: A Randomized Controlled Trial

**DOI:** 10.1097/ALN.0000000000004915

**Published:** 2024-01-19

**Authors:** Simone C. Jansen, Maarten van Lemmen, Erik Olofsen, Laurence Moss, Joseph V. Pergolizzi, Thomas Miller, Robert D. Colucci, Monique van Velzen, Philip Kremer, Albert Dahan, Rutger van der Schrier, Marieke Niesters

**Affiliations:** 1 Department of Anesthesiology, Leiden University Medical Center, Leiden, The Netherlands.; 2 Department of Anesthesiology, Leiden University Medical Center, Leiden, The Netherlands.; 3 Department of Anesthesiology, Leiden University Medical Center, Leiden, The Netherlands.; 4 Centre for Human Drug Research, Leiden, The Netherlands.; 5 Enalare Therapeutics Inc., Princeton, New Jersey; NEMA Research Inc., Naples, Florida.; 6 Enalare Therapeutics Inc., Princeton, New Jersey; Department of Pediatrics, Sidney Kimmel Medical College, Thomas Jefferson University, Philadelphia, Pennsylvania.; 7 NEMA Research Inc., Naples, Florida; Colucci & Associates, LLC, Newtown, Connecticut.; 8 Department of Anesthesiology, Leiden University Medical Center, Leiden, The Netherlands.; 9 Centre for Human Drug Research, Leiden, The Netherlands.; 10 Department of Anesthesiology, Leiden University Medical Center, Leiden, The Netherlands; PainLess Foundation, Leiden, The Netherlands.; 11 Department of Anesthesiology, Leiden University Medical Center, Leiden, The Netherlands.; 12 Department of Anesthesiology, Leiden University Medical Center, Leiden, The Netherlands; PainLess Foundation, Leiden, The Netherlands.

## Abstract

**Background:**

The use of anesthetics may result in depression of the hypoxic ventilatory response. Since there are no receptor-specific antagonists for most anesthetics, there is the need for agnostic respiratory stimulants that increase respiratory drive irrespective of its cause. The authors tested whether ENA-001, an agnostic respiratory stimulant that blocks carotid body BK-channels, could restore the hypoxic ventilatory response during propofol infusion. They hypothesize that ENA-001 is able to fully restore the hypoxic ventilatory response.

**Methods:**

In this randomized, double-blind crossover trial, 14 male and female healthy volunteers were randomized to receive placebo and low- and high-dose ENA-001 on three separate occasions. On each occasion, isohypercapnic hypoxic ventilatory responses were measured during a fixed sequence of placebo, followed by low- and high-dose propofol infusion. The authors conducted a population pharmacokinetic/pharmacodynamic analysis that included oxygen and carbon dioxide kinetics.

**Results:**

Twelve subjects completed the three sessions; no serious adverse events occurred. The propofol concentrations were 0.6 and 2.0 µg/ml at low and high dose, respectively. The ENA-001 concentrations were 0.6 and 1.0 µg/ml at low and high dose, respectively. The propofol concentration that reduced the hypoxic ventilatory response by 50% was 1.47 ± 0.20 µg/ml. The steady state ENA-001 concentration to increase the depressed ventilatory response by 50% was 0.51 ± 0.04 µg/ml. A concentration of 1 µg/ml ENA-001 was required for full reversal of the propofol effect at the propofol concentration that reduced the hypoxic ventilatory response by 50%.

**Conclusions:**

In this pilot study, the authors demonstrated that ENA-001 restored the hypoxic ventilatory response impaired by propofol. This finding is not only of clinical importance but also provides mechanistic insights into the peripheral stimulation of breathing with ENA-001 overcoming central depression by propofol.

## Abstract 2

The hypothesis that ENA-001 can restore the hypoxic ventilatory response during propofol infusion was tested in a double-blind, placebo-controlled crossover study in 12 normal adults. Subjects were randomized to receive placebo, low-dose ENA-001, and high-dose ENA-001 on three separate occasions, during each of which isohypercapnic hypoxic ventilatory responses were measured during placebo, low-dose propofol, and high-dose propofol infusions. ENA-001 was effective in restoring the propofol-impaired hypoxic ventilatory response. Using a pharmacodynamic model based on the interaction between oxygen sensitivity and carbon dioxide sensitivity, an ENA-001 concentration of 1.5 μg/ml was determined to be able to fully reverse the depressant effects of a steady state propofol concentration of 2 μg/ml.

## Visual Abstract:

**Figure:**
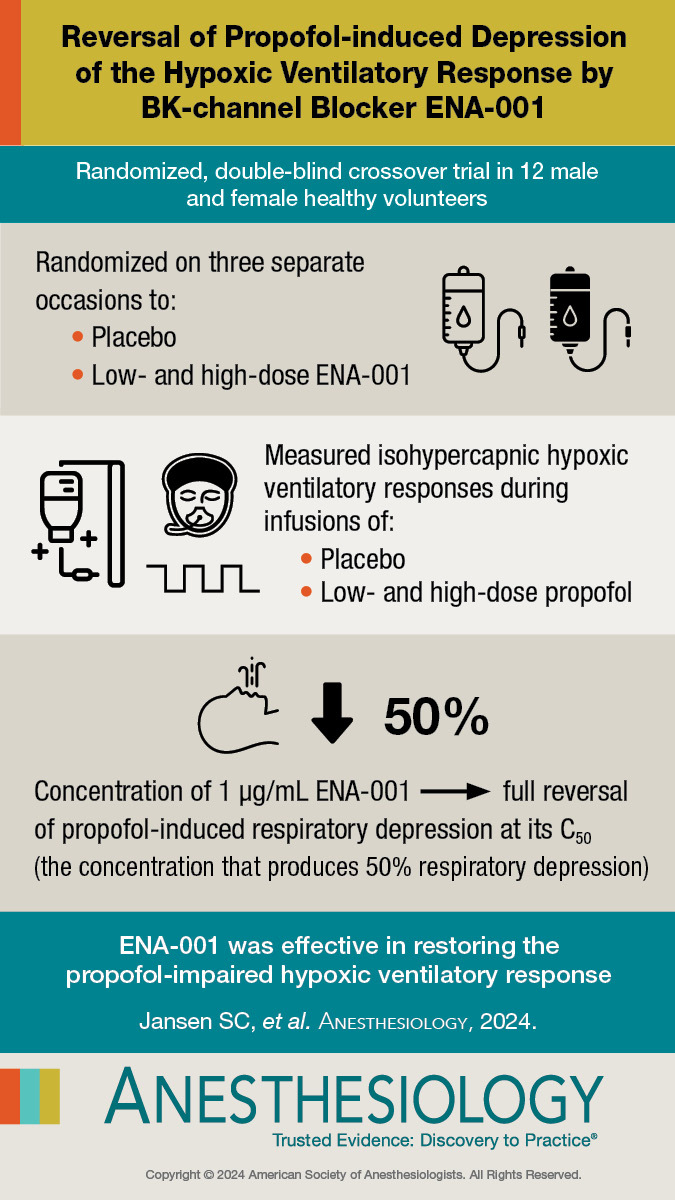

**Editor’s Perspective**

**What We Already Know about This Topic**

The carotid body is the main mammalian oxygen sensor and hypoxia triggers the hypoxic ventilatory response that restores pulmonary oxygen uptake

Propofol reduces the ventilatory response to hypoxia

ENA-001 is an agnostic respiratory stimulant that increases breathing activity by blocking calcium-activated potassium channels at the carotid bodies

**What This Article Tells Us That Is New**

The hypothesis that ENA-001 can restore the hypoxic ventilatory response during propofol infusion was tested in a double-blind, placebo-controlled crossover study in 12 healthy adults

Subjects were randomized to receive placebo, low-dose ENA-001, and high-dose ENA-001 on three separate occasions, during each of which isohypercapnic hypoxic ventilatory responses were measured during placebo, low-dose propofol, and high-dose propofol infusions

ENA-001 was effective in restoring the propofol-impaired hypoxic ventilatory response

Using a pharmacodynamic model based on the interaction between oxygen sensitivity and carbon dioxide sensitivity, an ENA-001 concentration of 1.5 μg/ml was determined to be able to fully reverse the depressant effects of a steady state propofol concentration of 2 μg/ml

Respiratory depression is a common side effect of anesthetics and analgesics that can lead to serious complications in perioperative patients.^1^ Since receptor-specific antagonists are not available for most of the nonopioid anesthetics, agnostic respiratory stimulants that increase breathing activity regardless of the underlying cause of respiratory depression are needed. ENA-001 (formerly known as GAL021) is an agnostic respiratory stimulant that increases breathing activity by blocking calcium-activated potassium channels (BK-channels) at the carotid bodies.^2–6^ In previous studies, it was demonstrated that ENA-001 can reverse respiratory depression induced by opioids in healthy volunteers and monkeys.^4–6^ Glomus type-1 carotid body cells express BK-channels and release neurotransmitters upon blockade of these channels. The excitatory neurotransmitters activate afferent nerves to the brainstem and subsequently increase breathing.^7,8^ The carotid body is the main oxygen sensor in the mammalian body, and hypoxia triggers the hypoxic ventilatory response (HVR), a lifesaving chemoreflex that restores pulmonary oxygen uptake. Although the exact mechanism of hypoxic sensing at the carotid bodies is not fully understood, potassium channels are believed to play a role in this process.^9^

Hypoxia is a common occurrence in postoperative patients and those undergoing sedation for minor procedures.^10^ The causes of hypoxia vary, but impairment of ventilatory control by anesthetic agents, such as inhalational and intravenous anesthetics, can be a significant contributing factor.^11^ There is ample evidence that inhalational anesthetics, at already subanesthetic concentrations, blunt the HVR at the carotid bodies. Intravenous agents such as propofol blunt the response within brainstem respiratory networks.^12–14^ Still, there is some evidence from small animal studies that at high dose, propofol (6 mg.kg^–1^.min^–1^) may have a direct depressant effect at the carotid bodies.^15^ To prevent or treat hypoxic events, agnostic respiratory stimulators may be utilized.^16^ This study aimed to assess the effect of ENA-001 on propofol-induced depression of the HVR in human volunteers. The results of this study will provide valuable information on ENA-001’s ability to overcome depression of the HVR induced within brainstem respiratory networks. We hypothesize that ENA-001 can restore the HVR during propofol infusion.

## Materials and Methods

### Ethics

The protocol was approved by the ethics committee BEBO Foundation for Assessment of Ethics of Biomedical Research (Assen, The Netherlands; date of approval, August 2, 2021) and the national competent authority, the Central Committee on Research Involving Human Subjects (The Hague, The Netherlands). All study procedures were conducted according to good clinical practice guidelines and adhered to the tenets of the Declaration of Helsinki. Before enrollment, all subjects gave written informed consent, after which their medical history was taken and a physical examination was performed. The study was performed from October 2021 until April 2022 at Leiden University Medical Center and the Center for Human Drug Research (Leiden, The Netherlands) and registered in the trial register of the Dutch Cochrane Center under identifier NL9692 (currently available at clinicaltrialregister.nl; registration date, August 22, 2021) with G. J. Groeneveld as principal investigator. No changes were made to the protocol or trial outcomes after trial commencement.

### Subjects

Healthy male and female volunteers age 18 to 55 yr with weight 55 to 100 kg, body mass index 18 to 39 kg/m^2^, and with normal vital signs observed during screening (body temperature between 35.5° and 37.5°C, systolic blood pressure between 90 and 150 mmHg, diastolic blood pressure between 40 and 95 mmHg, and pulse rate between 40 and 100 beats/min) were eligible for participation in the study. Both male and female participants had to use contraception during the trial and for 3 months thereafter in case they were sexually active or not surgically sterile. Exclusion criteria included a current diagnosis or history of a psychiatric disease, a history of substance abuse (including alcohol), smoking more than five cigarettes per week in the last year, positive urine drug screen at screening or on any of the test days, current or history of a medical (including allergies, malignancies) or surgical condition, motion sickness, participation in any other drug study in the last 3 months, a family history of malignant hyperthermia, high daily caffeine intake (*e.g.*, more than six coffee or tea beverages per day), and subjects with an anticipated difficult airway. All subjects were subjected to a urine drug screen and alcohol breath test upon screening and on the day before treatment on visits 1 to 3. In case of a positive test, the subjects were excluded from further participation in the study. The subjects were admitted to the clinical research unit (Center for Human Drug Research) from 1 day preceding to 1 day after the visit to the Anesthesia and Pain Research Unit at Leiden University Medical Center for the experimental tests.

### Study Design

**Fig. 1.**
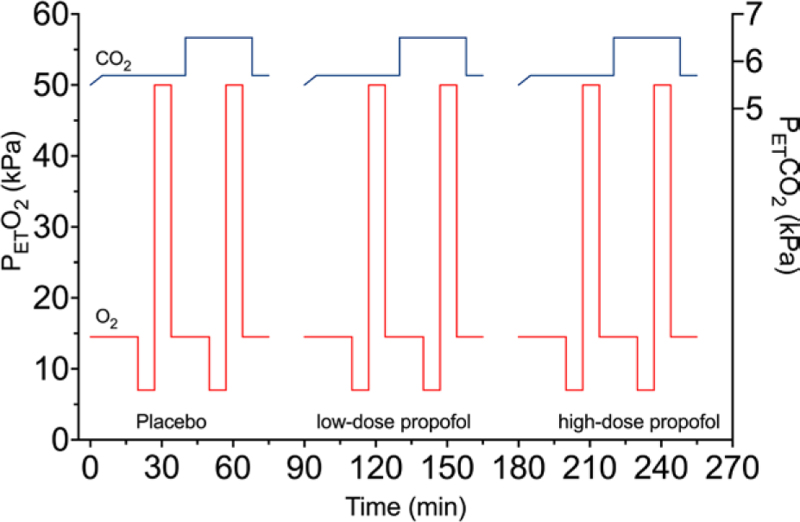
Schematic diagram of the hypoxic (*red lines*) and hypercapnic (*blue lines*) stimuli given on a single visit. The first set of stimuli is given during placebo infusion, the next during low-dose propofol infusion, and the last during high-dose propofol infusion. Each subject visited the research unit on three occasions. On one occasion, placebo was infused from time = 0 to time = 270 min; on another, low-dose ENA-001; and on another, high-dose ENA-001; these administrations were randomized. Petco_2_, end-tidal pressure of carbon dioxide.

The study had a double-blind, placebo-controlled crossover design. A schematic diagram of the protocol is given in figure 1. Each study subject underwent three study sessions on separate days. Each session was randomly assigned to placebo, low-dose ENA-001, or high-dose ENA-001. Each session started with the study drug alone, followed by low-dose propofol infusion, and finally high-dose propofol infusion. At each of the study steps or conditions (no propofol, low-dose propofol, high-dose propofol), we tested the ventilatory response to hypoxia (reduced inspired oxygen fraction such that the arterial oxygen saturation is about 80%) at both low and high end-tidal PCO_2_. We report on the pharmacokinetic–pharmacodynamic modeling of the effect of ENA-001 on the depression of the HVR (primary endpoint) and give a short statistical analysis of the raw data (secondary endpoint).

### Randomization, Allocation, and Blinding

Subjects who successfully completed the screening and had given written informed consent were given a subject number. All subject numbers were randomized for ENA-001 treatment using a computer-generated randomization list (with six possible sequences for placebo and low- and high-dose ENA-001). The randomization code was made available to the pharmacy and an unblinded physician of the department of anesthesiology to set the ENA-001 infusion pump but otherwise kept confidential until the study was completed and the data were locked.

### Treatment

Intravenous propofol and ENA-001 were prepared in syringes by the Leiden University Medical Center pharmacy with the ENA-001 dispensed in unmarked sterile infusion bags (except for study name, subject number, and study visit). The drugs were infused using Infusomat Space infusion pumps (ref. 87133050, Braun, Germany). The ENA-001 infusion scheme was based on the subject’s weight and mirrored the dosing scheme of a previous study:^4,5^ low-dose 33.3 µg · kg^–1^ · min^–1^ for 10 min followed by a continuous infusion of 6.7 µg · kg^–1^ · min^–1^ until the end of the study session, and high-dose 33.3 µg · kg^–1^ · min^–1^ for 20 min followed by a continuous infusion of 18.3 µg · kg^–1^ · min^–1^. An unblinded physician not involved in the study set the infusion pump to the correct infusion rate, and the infusion rate was not visible to the research team. Propofol infusion was not blinded and was set at 239 µg · kg^–1^ · min^–1^ for 3 min, followed by 0 µg · kg^–1^ · min^–1^ for 6 min and 24 µg · kg^–1^ · min^–1^ for 61 min (low-dose phase), a subsequent transition dose for 15 min of 47 µg · kg^–1^ · min^–1^, and subsequently 239 µg · kg^–1^ · min^–1^ for 3 min, followed by 0 µg · kg^–1^ · min^–1^ for 6 min and 44 µg · kg^–1^ · min^–1^ for 61 min (high-dose phase).

### Respiratory Testing

We used the dynamic end-tidal forcing technique to induce steps in end-tidal partial pressures of carbon dioxide (Petco_2_) and end-tidal PO_2_ so that we could study the ventilatory response to isocapnic hypoxia at two levels of Petco_2_.^13,14^ To that end, the subjects breathed through a mask covering their nose and mouth that was connected to a pneumotachograph and pressure transducer system (Hans Rudolph Inc., USA) to measure ventilation. Additionally, we measured inspired and expired gas concentrations at the mouth using a Masimo Root ISA OR plus capnograph (Masimo, USA). The subjects inhaled a gas mixture that was delivered by the second generation Leiden gas mixer controlled by ACQ/RESREG software. The system (software and hardware) is custom-built (Leiden University Medical Center) and allows collection of ventilation and end-tidal gas concentrations on a breath-to-breath basis and enables imposing variations in inspired gas concentrations to achieve the desired end-tidal Po_2_ and Petco_2_, independent of the ventilatory response. In this study, we applied the following end-tidal gas concentration sequence (fig. 1): end-tidal PO_2_ 13.5 kPa (101 mmHg) for 7 to 10 min, 5.8 kPa (44 mmHg) for 5 min (hypoxia at low PCO_2_), 50 kPa (375 mmHg) for 5 min, 13.5 kPa (101 mmHg) for 7 to 10 min, 5.8 kPa (44 mmHg) for 5 min (hypoxia at high PCO_2_), 50 kPa (375 mmHg) for 5 min, and 13.5 kPa (101 mmHg) for 7 to 10 min, while the Petco_2_ was kept at 0.3 kPa (2 to 3 mmHg) above resting values for 18 min and 1.3 kPa (10 mmHg) above resting for the remaining time (sequence duration, 46 min); the low end-tidal PO_2_ values correspond with oxygen saturation levels of 80 ± 2%. This sequence was performed three times on each study day, first during infusion of placebo, next during infusion of low-dose propofol, and finally during infusion of high-dose propofol, and always during the continuous infusion phase of drug administration. ENA-001 or placebo infusion started 30 min before respiratory measurements. The hyperoxic episodes are introduced to counteract any residual effects of 5-min hypoxia.^17^

We tested two oxygen levels: end-tidal PO_2_ 13.5 kPa (101 mmHg) and 5.8 kPa (44 mmHg). It is our ample experience with hypoxic studies that the former corresponds to an oxygen saturation of 97 to 100%, the latter to a saturation of 80 ± 2%.^9,13,17,18^ We did so to induce a brisk hyperventilatory response. Because the relationship between arterial oxygen saturation and ventilation is linear,^9,18^ additional hypoxic levels are not needed to get a reliable estimate of the HVR. Most studies on the effect of drug that we performed do apply hypoxia levels with inspired fractions of 0.05 to 0.058 for 5 min as it is considered a safe level of hypoxia in healthy volunteers.^18^ We have safety rules in place in our laboratory that mandate the administration of 100% oxygen when saturation levels fall lower than 74%. In this study, subjects’ safety rules were not required in any of the subjects.

### Blood Sampling

Arterial blood samples (2 ml) for ENA-001 measurement were collected at time = 0 (the start of ENA-001 infusion) and 5, 10, 15, 20, 25, 30, 60, 90, 115, 145, 175, 200, 230, 260, 270, 280, and 300 min. Arterial blood samples (2 ml) for propofol measurements were obtained at time = 0 and 145, 175, 200, 230, 260, 270, 280, and 300 min. Both drugs were measured in 50 µl K2-EDTA plasma by Ardena Bioanalysis BV (The Netherlands) using validated liquid chromatography tandem mass spectrometry assays. For ENA-001, the assay was validated over the concentration range of 0.25 to 4,000 ng/ml with a maximum bias of 15% and coefficient of variation of 15%; for propofol, the assay was validated for the concentration range 10 to 40,000 ng/ml with a maximum bias of 15% and coefficient of variation of 15%.

### Safety

Throughout the visit to the research unit, blood pressure, heart rate, and oxygen saturation were measured continuously. In case of respiratory adverse events beyond the scope of the study, dosing could be reduced, supplemental oxygen could be given, and the subjects could be ventilated by mask. Such events included end-tidal PCO_2_ greater than 9 kPa (60 mmHg) for at least 3 min or less than 3.3 kPa (25 mmHg) for at least 2 min, oxygen saturation measured by pulse oximetry less than 90% for at least 1 min during breathing of a normoxic gas mixture, or a respiratory rate less than 4 min^–1^ for at least 1 min.

### Data Analysis

The pharmacokinetics and pharmacodynamics of ENA-001 and propofol were analyzed with NONMEM 7.5.1 (Icon Plc., Ireland) using a population approach.

### ENA-001 and Propofol Pharmacokinetics

The pharmacokinetic data were analyzed using a two-compartmental model. The models were fitted to the data assuming linear scaling with weight using the conditional estimation with interaction method. Diagnostic plots were inspected for outliers.

### Oxygen and Carbon Dioxide Pharmacokinetics

**Fig. 2.**
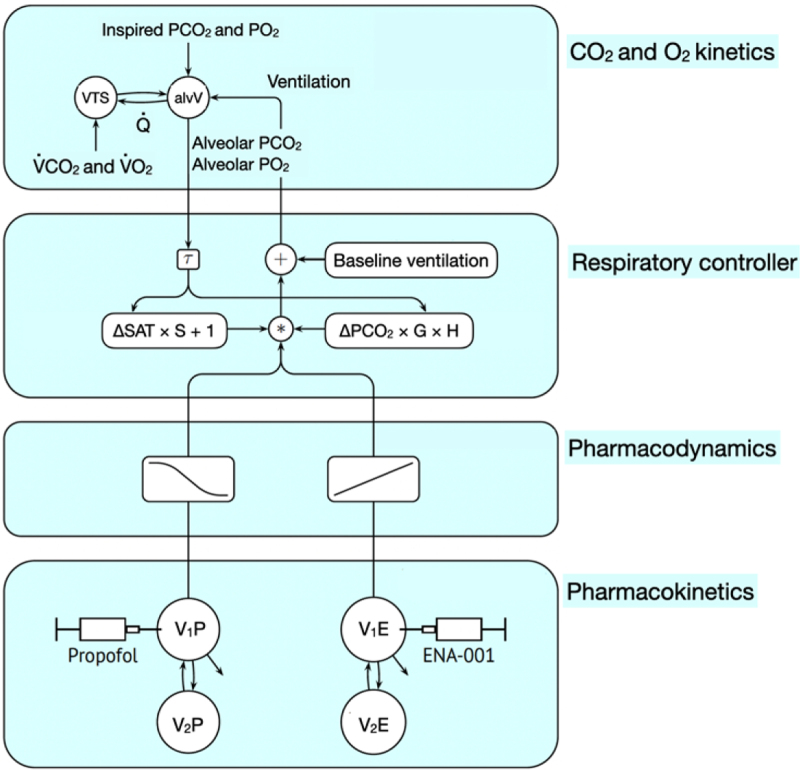
Schematic diagram of the pharmacokinetic and pharmacodynamic model. The model has four distinct parts. Part 1 (*bottom*) is the part that describes the pharmacokinetics of propofol and ENA-001. Part 2 (*second from bottom*) describes the pharmacodynamics of propofol with an inhibitory sigmoid Emax model and ENA-001 with a power model (approximated by a linear function). Propofol reduces hypoxic/hypercapnic ventilation, while ENA-001 stimulates the propofol-induced depression of hypoxic or hypercapnic ventilation. Part 3 (*second from top*) is the respiratory controller where oxygen and carbon dioxide sensitivity interact in a multiplicative fashion at *. Part 4 (*top*) describes oxygen and carbon dioxide kinetics. VTS, tissue volume; alvV, alveolar ventilation; VCO_2_, carbon dioxide production; τ, time constant; VO_2_, oxygen consumption; ΔSAT, difference between baseline and effect-site oxygen saturation; S, hypoxic ventilatory sensitivity; ΔPCO_2_, difference between alveolar and baseline PCO_2_; G, hypercapnic ventilatory sensitivity; H, hinge function; V_1_P and V_2_P, volumes of the first and second propofol pharmacokinetic compartments; V_1_E and V_2_E, volumes of the first and second ENA-001 pharmacokinetic compartments.


Carbon dioxide and oxygen kinetics (fig. 2) were modeled *via* the following differential equations^19,20^:

$$\begin{array}{l} V_{AL}\cdot \frac{d\;\left(alveolar\;Px\right)}{dt}\;\\ ={\dot{V}}_{A}\cdot \left(inspired\;Px-alveolar\;Px\right)\;\\ +\lambda_{0}\cdot \dot{Q}\cdot \left(mixed\;venous\;Cx-\;alveolar\;Cx\right), \end{array}$$ (1)

and


$$\begin{array}{l} V_{TS}\cdot \frac{d\left(mixed\;venous\;Cx\right)}{dt}\;\\ =\dot{Q}\cdot \left(alveolar\;Cx-mixed\;venous\;Cx\right)\pm \lambda_{2}\cdot \dot{M},\; \end{array}$$ (2)

where V_AL_ is alveolar volume, V̇ _A_ is alveolar ventilation, *x* is either oxygen or carbon dioxide, alveolar gas partial pressure (alveolar P) is assumed to be equal to arterial pressure, C is content, alveolar gas content (alveolar C) is assumed to be equal to arterial content, V_TS_ the apparent tissue volume (*i.e.*, the whole body), $\dot{Q}$ cardiac output, λ_0_ ≈ 1.2 is a constant describing the conversion of standard temperature, pressure, dry (STPD) to body temperature and pressure saturated (BTPS), and λ_2_ = 100 is a unit conversion between volume of gas in air and in blood. The dependencies of the pressure and blood content variables on time have been excluded for the sake of legibility. As the equations are similar for carbon dioxide and oxygen, Ṁ denotes either carbon dioxide production or oxygen consumption, respectively. Parameters were fixed to literature values, except V_TS_ to allow for a variable delay between a change in inspired and alveolar partial pressures. The initial change in the alveoli is very fast (within 1 min), but incorporating the first differential equation avoids solving the steady state equation, which is not easily implemented because of the nonlinear relationship between oxygen pressure and blood content. Note that the unit for content is milliliter of gas per 100 ml of blood.

Blood chemistry was simplified as much as possible by assuming the following relationships between pressure and blood content:


$$\begin{array}{l} alveolar\;CO_{2}\;content=\frac{alveolar\;PCO_{2}}{\lambda_{1}} \end{array}$$ (3)

$$\begin{array}{l} P=alveolar\;PO_{2} \end{array}$$ (4)

$$\begin{array}{l} SO_{2}=1/\left(1+\frac{23400}{P\cdot \left(P\cdot P+150\right)}\right) \end{array}$$ (5)

$$\begin{array}{l} alveolar\;CO_{2}=20.85\cdot SO_{2}+0.003\cdot P\; \end{array}$$ (6)

where λ_1_ = 0.115 is a linear approximation of the solubility of carbon dioxide in blood, and the equation for blood saturation, SO_2_, is an approximated oxygen saturation in arterial blood.^21^

### Ventilatory Controller

Minute ventilation (fig. 2) was assumed to be approximately linearly related to alveolar PCO_2_ and SO_2_:


$$\begin{array}{l} \begin{array}{l} V_{E}=baseline\;V_{E}-G\cdot H\left(0\right)\;\\ +[S\cdot (baseline\;SO_{2}-effect\;site\;SO_{2})+1]\;\\ \cdot G\cdot H\left(effect\;site\;PCO_{2}-alveolar\;PCO_{2}\;at\;baseline\right),\; \end{array} \end{array}$$ (7)

with


$$\begin{array}{l} H\left(x\right)=\;\delta \cdot \log\left(1+\exp\left(\frac{x}{\delta }\right)\right),with\;\delta =\;0.1, \end{array}$$ (8)

and H(x) is the “hinge” function,^22^ allowing for a nonlinearity around baseline PCO_2_ (*i.e.*, the Hinge function describes the ventilatory transition from normocapnia to hypercapnia; see also Hellinga *et al.*^20,23^) with x = (effect-site PCO_2_ – baseline PCO_2_) and H(x) = 0 when x < 0 and H(x) = x when x > 0. G · H(0) was subtracted from baseline ventilation, because it is not exactly equal to zero at baseline PCO_2_ (x = 0). Effect site SO_2_ and PCO_2_ denote the blood oxygen saturation and carbon dioxide pressure at the respiratory controller, where the former was calculated from the effect-site Po_2_. The effect-site gas pressures were assumed to be delayed with respect to the alveolar/arterial pressures *via* time constant τ. S is oxygen sensitivity and G carbon dioxide sensitivity.

The multiplication between the carbon dioxide and oxygen dependent terms in equation 7 (fig. 2) allows for the interaction of carbon dioxide and oxygen on ventilation (at hypercapnia the hypoxic sensitivity increases), an effect attributed to the carotid bodies.

The solution of the differential equations requires initial conditions for alveolar PCO_2_ and PO_2_. From these, initial values for alveolar carbon dioxide and oxygen content were calculated from the equations above, and initial conditions for venous carbon dioxide and oxygen content were calculated from the steady state solutions of the differential equations for the tissues. The difference between minute ventilation and alveolar ventilation, dead space ventilation, was estimated (V_D_). The three output variables alveolar PCO_2_, alveolar PO_2_, and minute ventilation were simultaneously fitted. The nine parameters to be estimated were the tissue storage volume for carbon dioxide and oxygen, alveolar PCO_2_ and PO_2_ at baseline, τ, baseline ventilation, dead space ventilation, G, and S. The following parameters were fixed to their physiologic or pharmacologic values: cardiac output 5 l/min, carbon dioxide production 200 ml/min, oxygen consumption 250 ml/min, alveolar volume 3 l, and hematocrit = 0.4 with oxygen content at 100% saturation 20.85 ml per 100 ml blood.^24^

### Pharmacodynamics

Propofol and ENA-001 were assumed to have a depressant and excitatory effect on hypoxic or hypercapnic ventilation, respectively (fig. 2). Therefore, the multiplicative term in equation 7 (asterisk in fig. 2) was multiplied with the following empiric function of the effect-site propofol and ENA-001 concentrations, C_E_propofol (C_E_P) and C_E_ENA-001 (C_E_E):


$$\begin{array}{l} F\left(C_{E}P,\;C_{E}E\right)=\frac{1+0.5\cdot \left(\frac{C_{E}E}{C_{50}E}\right)}{1+{\left(\frac{C_{E}P}{C_{50}P}\right)}^{\gamma }} \end{array}$$ (9)

where C_50_P and C_50_E are the concentrations of propofol and ENA-001 that give 50% depression and 50% reversal, respectively, and γ a shape parameter. Propofol blood–effect-site half-time was assumed to be 2.5 min, and ENA-001 blood–effect-site half-time 0 min.

From equation 9, it follows that ENA-001 counteracts propofol depression when F = 1, so when


$$\begin{array}{l} CE=2\;\cdot C_{50}E\cdot {\left(\frac{C_{E}P}{C_{50}P}\right)}^{\gamma }, \end{array}$$ (10)

where CE is the ENA-001 concentration and CP the propofol concentration. The subscript E denotes effect-site concentration. The 3 parameters to be estimated are C_50_P, C_50_E, and γ, bringing the total number of parameters to be estimated to 12.

### Statistical Analysis

No formal sample size analysis was performed. We consider the current sample of 12 to 14 subjects a convenience sample, and mimicked our earlier study on the influence of the same doses of ENA-001 as used in the current study on alfentanil-induced respiratory depression. In that study,^4,5^ 12 subjects were sufficient to detect a significant reversal of respiratory depression (increase in ventilation relative to placebo 6.1 l/min at an alfentanil plasma concentration of 40 to 50 ng/ml). In the current study, we anticipated a similar stimulatory effect of ENA-001 in 12 to 14 healthy and young subjects.

The primary endpoint was the PK/PD analysis (*i.e.*, the steady state or effect-site ENA-001 concentration to increase the propofol-depressed ventilatory response by 50%). The PK/PD models were fitted to the data in NONMEM using a sequence of its estimation steps: iterative two-stage for initial estimates, stochastic approximation expectation maximization for parameter estimation, importance sampling for objective function evaluation, and Bayes for assessment of standard errors of the model parameters and derived parameters (for example, eq. 10). With 12 subjects and three visits, the numbers of interindividual (ω^2^) and interoccasion (ν^2^) variance terms were limited and assigned to the most plausible parameters; η-shrinkages were inspected to check for validity of this approach. Visual predictive checks were run using Pearl speaks NONMEM (https://uupharmacometrics.github.io/PsN/) with default settings and stratification on dose level for ENA-001 kinetics and data type: alveolar PCO_2_, alveolar Po_2_, and minute ventilation. *P* values < 0.01 were considered significant.

Full descriptive analyses are published elsewhere.^25^ Here we present a secondary analysis on the HVRs at low- and high-dose ENA-001 *versus* placebo. The data were analyzed with a mixed model with fixed factors treatment (ENA-001), condition (propofol), and treatment by condition and random factors subject, subject by treatment, and subject by condition. If a significant treatment effect was detected, the contrasts low-dose ENA-001 *versus* placebo and high-dose ENA-001 *versus* placebo were calculated within the model. If the interaction term treatment by condition was not significant, no further comparisons were performed. For the descriptive analyses, *P* values < 0.05 were considered significant, without type 1 error control. The analysis was performed in SAS for Windows (SAS Institute Inc., USA).

## Results

A total of 14 subjects were randomized. Twelve subjects completed all three visits without serious side effects; two subjects withdrew from the study on the first study day because of anxiety in one subject occurring during the first hypoxic test, before any drug administration and restlessness, and anxiety during high-dose propofol infusion but before hypoxic testing in another subject. Both subjects were exposed to high-dose ENA-001. No serious adverse events related to study medication were observed. Predominantly observed among treatment-related adverse events was infusion site pain, lasting approximately 20 min (n = 8 for low-dose ENA-001 and n = 12 for high-dose ENA-001), with no discernable trend among other adverse events. The characteristics of the 12 subjects are given in table 1; their data were used in the PK/PD and statistical analyses.

**Table 1.**
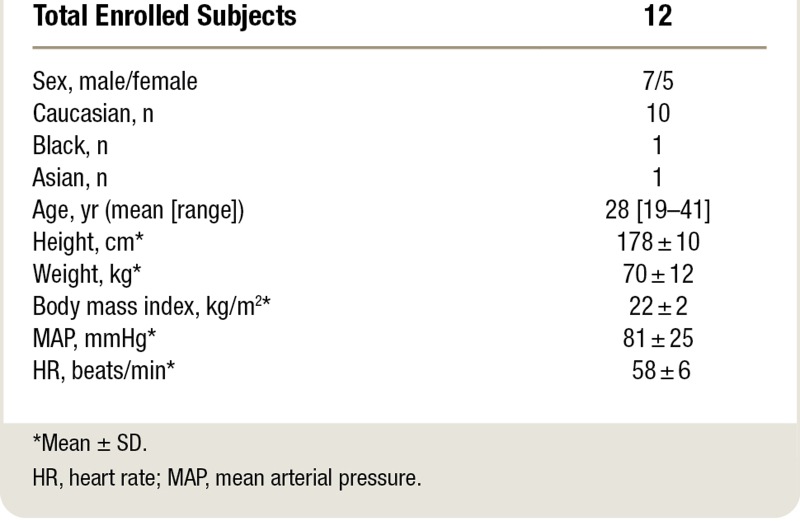
Demographic Data of Participants

**Fig. 3.**
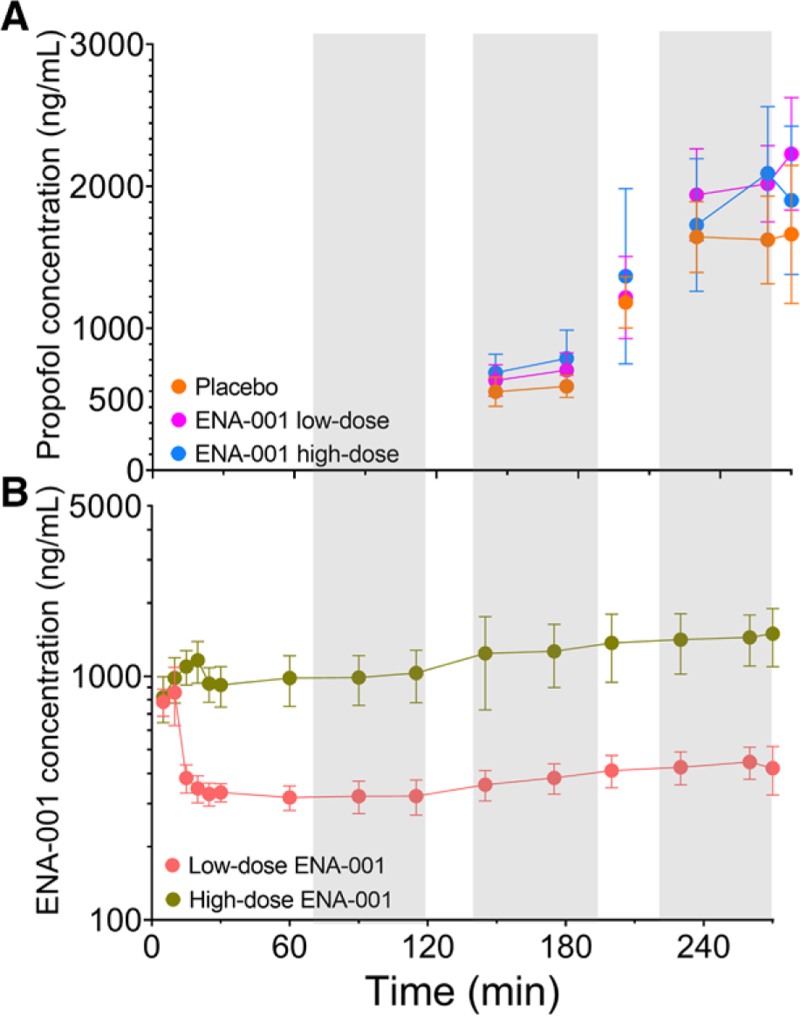
Plasma concentrations of propofol (*A*) and ENA-001 (*B*) during each study visit. The *gray areas* depict the time of the hypoxic and hypercapnic stimuli. Data are mean ± SD.

**Fig. 4.**
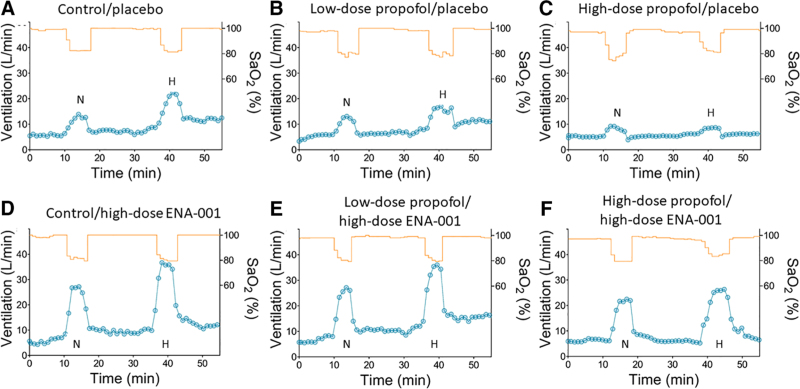
Effect of placebo (*A* to *C*) and high-dose ENA-001 (*D* to *F*) on hypoxic ventilatory responses of subject 006. In each, the responses to hypoxia at normocapnia (N) and hypercapnia (H) are given (*left* to *right*). *A* and *D*, Control response (no propofol) with (*D*) the ENA-001 effect. *B* and *E*, Low-dose propofol with (*E*) the ENA-001 effect. *C* and *F*, High-dose propofol with (*F*) the ENA-001 effect. The *orange lines* depict oxygen saturation. Each *blue circle* depicts a 1-min ventilation average. Sao_2_, arterial oxygen saturation.

**Fig. 5.**
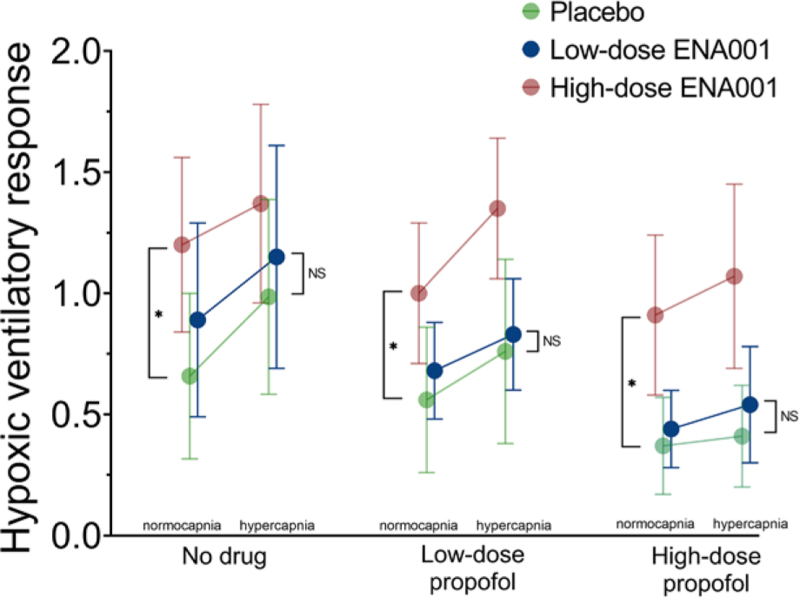
Average hypoxic ventilatory responses at normocapnia and hypercapnia during no drug infusion and low- and high-dose propofol infusion with treatments placebo (*green*), low-dose ENA-001 (*blue*), and high-dose ENA-001 (*red*). Treatment effect was significant for high-dose ENA-001 *versus* placebo (*P < 0.0001) but not for low-dose ENA-001 *versus* placebo (*P* = 0.090). Data are mean ± 95% CI.

The mean ± SD ENA-001 and propofol concentration profiles during respiratory testing are presented in figure 3. An example of the effect of placebo and high-dose ENA-001 on HVRs of a single subject is given in figure 4. The top three panels (fig 4., A to C) depict the responses at normocapnia and hypercapnia during no drug (control; fig. 4A), low-dose propofol (fig. 4B), and high-dose propofol (fig. 4C) without any ENA-001 infusion. It shows the depressant effect of propofol on the HVR with a control (no drugs given) response at normocapnia and hypercapnia of 0.44 and 0.72 l .min^–1^.%^–1^, respectively (fig. 4A), which is reduced to 0.17 and 0.15 l .min^–1^ .%^–1^ during high-dose propofol infusion (fig. 4C). During infusion of high-dose ENA-001, the HVR increased to 1.12 and 1.28 l .min^–1^ %^–1^ at normocapnia and hypercapnia, respectively (no propofol given; fig. 4D), while during high-dose propofol infusion, the responses were 0.88 and 1.54 l .min^–1^ .%^–1^ (fig. 4F) at low and high carbon dioxide levels, respectively. The responses at high-dose propofol (fig. 4F), in this particular subject, were greater than those observed without any drug infused (fig. 4A). The mean responses ± 95% CI at each treatment level are given in figure 5. We observed a significant ENA-001 treatment effect (*P* < 0.0001) and a significant condition effect (*P* < 0.0001), while the interaction term treatment by condition was not significant (*P* = 0.402). *Post hoc* analysis revealed a significant effect of high-dose ENA-001 *versus* placebo (*P* < 0.0001) but not low-dose ENA-001 *versus* placebo (*P* = 0.090). Figure 5 further depicts the multiplicative effect of hypercapnia on the HVR, particularly during no drug and low-dose propofol.

**Fig. 6.**
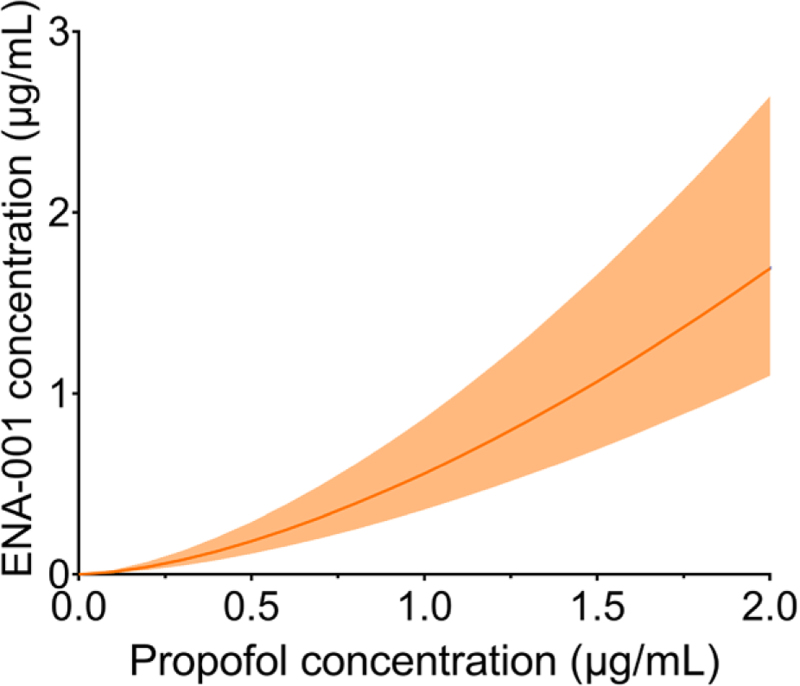
ENA-001 concentration (*y-axis*) that fully reverses the depression of the hypoxic/hypercapnic ventilatory response induced by a specific propofol concentration (*x-axis*). The data are ± 95% CI.

Pharmacokinetic parameter estimates are given in Supplemental Table 1 (https://links.lww.com/ALN/D449). In Supplemental Figure 1 (https://links.lww.com/ALN/D449), the measured ENA-001 data and individual data fits are presented for low- and high-dose ENA-001 (A and B) and goodness-of-fit plots (C and D). These graphs indicate that the PK model adequately described the ENA-001 data. The pharmacodynamic parameter estimates are presented in table 2 for the 12 estimated parameters and their variability estimates. The steady state concentration propofol that reduced the HVR by 50% was 1.47 ± 0.20 µg/ml, and the steady state ENA-001 concentration to increase the depressed ventilatory response by 50% was 0.51 ± 0.04 µg/ml. For validation of the standard errors of the estimate, they were compared with those from the importance sampling step and were all of the same order of magnitude. For the two important potency parameters, the 95% CIs were determined using the Pearl speak NONMEM “llp” procedure. These were 1.03 to 2.00 and 0.41 to 0.63 µg/ml for the C_50_ of propofol and ENA-001, respectively. At their C_50_ values, the combined effect of both drugs resulted in an overall 25% reduction of the HVR. A steady state concentration of 1 µg/ml ENA-001 was needed for full reversal of the propofol effect at its C_50_ (fig. 6). Goodness-of-fit plots are presented in Supplemental Figure 2 (https://links.lww.com/ALN/D449) for the three simultaneously fitted parameters: alveolar PCO_2_, alveolar Po_2_, and minute ventilation. These diagnostic graphs indicate that the data were well-described by the physiologic pharmacodynamic model depicted in figure 2. Population-predicted ventilatory responses to hypoxia and hypoxia/hypercapnia are given in Supplemental Figure 3 (https://links.lww.com/ALN/D449) for all nine conditions tested. These graphs indicate that at high-dose propofol, the stimulatory effect of high-dose ENA-001 caused the return of the ventilatory response to placebo levels without any drug (compare green line of the high-dose propofol data with the red line of the placebo data).

**Table 2.**
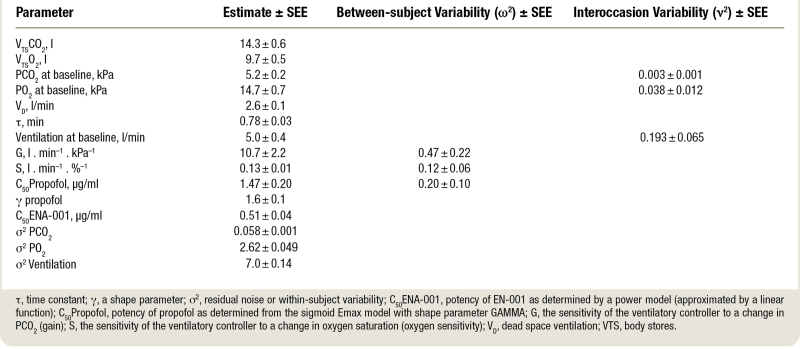
Pharmacodynamic Model Estimates

## Discussion

In the current study, we quantified the effect of the BK-channel blocker ENA-001 on propofol-induced depression of the HVR. Our findings demonstrate that ENA-001 was effective in restoring the propofol-impaired HVR, as shown in figure 5. Additionally, we determined that to fully reverse the depressant effects of propofol at a steady state concentration of 2 µg/ml, an ENA-001 concentration of 1.5 µg/ml was required, as illustrated in figure 6. These results support our hypothesis that ENA-001 can reverse the effect of centrally acting depressants on the HVR during normocapnia and hypercapnia over the propofol concentration range tested (0 to 2,000 ng/ml).

### Propofol-induced Respiratory Depression

We and others showed that propofol has a negative impact on metabolic ventilatory control.^13,14,26^ Particularly, it reduces the ventilatory response to hypoxia, and can lead to hypercapnia, bradypnea, or apnea at high dose.^13,14,26^ Given its widespread use in anesthesia, in procedural sedation, and during surgery under regional anesthesia, it is important to understand its effects on ventilatory control and examine the ability to reverse or prevent such adverse effects.

In a previous study in healthy volunteers, we demonstrated that propofol, at measured plasma concentrations of 0.5 to 1.3 µg/ml and Bispectral Index values around 70, primarily affects ventilatory control within the central chemoreflex loop at the central chemoreceptors.^14^ We observed that propofol reduced the gain of the slow (central chemoreflex) component of the ventilatory response to a multifrequency binary carbon dioxide sequence without affecting the gain of the fast (peripheral chemoreflex) component or the response dynamics. This suggests that propofol does not affect the peripheral chemoreceptors at the carotid bodies, as supported by a study that showed that sudden inhalation of 100% oxygen rapidly reduced ventilation in propofol-anesthetized patients.^26^ Still, there are both animal and human data that show that propofol blunts the HVR.^13,15,27^ While human studies were not able to detect the site of action of propofol, Ponte and Sadler showed that at high rates of propofol infusion (6 mg/min), the response to hypoxia but not to potassium was abolished in rats and rabbits as measured from single chemoreceptor fiber afferent output measured in the sinus nerve just proximal from its junction with the glossopharyngeal nerve.^15^ At lower doses (2 mg/min), depression of chemosensitivity was still observed but less marked. These data indicate that a direct effect of propofol on the carotid body may arise at high doses of propofol, higher than used in the current study. Given the above, at the propofol concentrations measured in our study (up to 2.2 µg/ml and Bispectral Index values 50 to 60), a dominant effect at the carotid bodies seems unlikely, while we cannot rule out an effect at pathways common to the peripheral and central chemoreflex loops, such as brainstem respiratory centers, phrenic nerve motor pool, lung, or diaphragm. We therefore attribute the reduced HVR observed in our current study to an effect at the central chemoreceptors, the brainstem respiratory centers, or efferent ventilatory motor pathways, which collectively depress the HVR.

In this context, it is important to note that the stimulatory effect of ENA-001 in our study is thus thought to originate at the intact carotid bodies. Additional research is needed to ascertain the ENA-001 effect when ENA-001 is administered in conjunction with drugs used in anesthesia that target the carotid bodies (see section “ENA-001 Effect on Ventilatory Control”).

### ENA-001 Effect on Ventilatory Control

In rats, ENA-001 increases sinus nerve output in a dose-dependent manner, but this effect disappears upon carotid body denervation.^6^ In mice that lack crucial subunits of the BK-channel (*Slo*^–/–^ mice), the effect of ENA-001 was significantly reduced, indicating that the molecular target for ENA-001 is the BK-channel expressed on type 1 cells of the carotid bodies, which are the peripheral chemoreceptors.^6^ Our current study confirms these findings by showing that ENA-001 enhances the HVR during placebo and propofol infusions, which we relate to its effect at the carotid body BK-channels.

While it was previously believed that peripheral stimulation may not be sufficient to overcome respiratory depression arising at central sites,^5,28^ our current study challenges this idea by demonstrating the efficacy of ENA-001 in overcoming central respiratory depression without any signs of ceiling over the measured propofol concentration range (0 to 2,000 ng/ml). However, as mentioned above, it should be noted that ENA-001 has only been studied under the condition of an intact carotid body function. Various drugs used in anesthesia and in the intensive care unit impair the carotid body response to hypoxia and hypercapnia. For instance, low-dose (less than 0.1 to 0.2%) halothane, isoflurane, and sevoflurane have been shown to severely blunt the HVR by an effect exclusively at the carotid bodies, and low-dose dopamine infusion behaves similarly.^11,12,29,30^ Additionally, carotid body function may be affected by various illnesses such as atherosclerosis or diabetes mellitus. Thus, further research is needed to determine whether ENA-001 can restore carotid body response to hypoxia under these conditions.

### Population Pharmacokinetic–Pharmacodynamic Model

In this study, we investigated the effects of propofol and ENA-001 on the ventilatory response to hypoxia at the background of low and high levels of hypercapnia. To describe the effect of ENA-001 on carotid body activity, we developed a pharmacodynamic model based on the interaction between oxygen sensitivity (S) and carbon dioxide sensitivity (G), represented by the term S × G in equation 7, which we termed the HVR for practical purposes. The model adequately described the data, and our model parameter estimates (table 2) were consistent with previous studies.^14,19,20,31^ In concert, this supports our assumption that modeling ENA-001 effect at the oxygen–carbon dioxide multiplicative site within the carotid bodies was physiologically relevant.

The term S × G equals approximately 1.0 at baseline; propofol reduced this term by 50% at a steady state concentration of 1.5 µg/ml, which was restored by ENA-001 at a steady state concentration of 0.5 µg/ml by 50% (S × G = 0.75). The concentration ENA-001 required to fully reverse the blunted HVR caused by propofol at a steady state propofol concentration of 2 µg/ml was 1.6 µg/ml (S × G = 1.0). Our results indicate that ENA-001 can reverse the effect of a centrally acting respiratory depressant on the HVR within the tested range of propofol concentrations (0 to 2 µg/ml).

### Study Limitations

We acknowledge some limitations in our study. This study was performed in a small data set, and no formal power analysis was conducted for the pharmacokinetic or pharmacodynamic analysis, which was a secondary analysis of the data. The pharmacodynamic model we used to assess the effect of ENA-001 on the interaction between oxygen and carbon dioxide may not have fully captured the multiplicative process physiologically, and is best considered a fair approximation. Additionally, our study was conducted in a highly controlled experimental setting with a small sample size of healthy young adults and with fixed levels of hypoxia and hypercapnia, which may not reflect the variability of patients who receive multiple drugs and may have complex comorbidities. Furthermore, our study only examined the effect of ENA-001 on one anesthetic agent, propofol, while multiple drugs are retained in the body after general anesthesia or during procedural sedation. However, our study certainly has some clinical relevance as it tested a common clinical observation in postoperative and procedural patients, *i.e.*, hypoxia combined with hypercapnia. Additionally, our study serves as a mechanistic proof of concept and provides a model for future studies examining the effect of other stimulants, such as doxapram, another potassium channel blocker that stimulates breathing *via* an effect at the carotid bodies.^32^ It is important to note that our findings should be confirmed in additional studies, including those in postoperative patients or patients who undergo a minor procedure under sedation, and those examining the effect of ENA-001 in the presence of drugs that impair carotid body function. Moreover, the current results related to the measured plasma concentrations of ENA-001 and propofol, and extrapolations beyond the measured data should be performed with caution.^5^ Further studies are needed to address the effect of higher doses of ENA-001 than studied here on higher concentrations of propofol than applied here. Finally, in this crossover study, we cannot exclude some carryover effects. The time between study days, however, was ample, and within-day effects of the previous propofol dose were considered in the PK/PD analyses. Overall, we argue that carryover effects were of limited importance in the outcome of our study.

### Conclusions

Our study demonstrates that ENA-001 can effectively restore the HVR under conditions of central respiratory depression due to propofol administration. Our mechanistic model suggests that ENA-001 acts on the BK-channels in the carotid body, specifically *via* the oxygen–carbon dioxide multiplicative component of the HVR. Our findings, supported by previous studies, indicate that ENA-001 has potential as an agnostic respiratory stimulator for preventing or treating respiratory depression from drugs that depress ventilation at central sites, *i.e.*, within the brainstem respiratory networks.

### Research Support

Financial support was obtained from institutional and/or departmental sources and from Center of Human Drug Research, Leiden, The Netherlands.

### Competing Interests

In the last 36 months, Dr. Dahan received consultancy fees from Enalare Therapeutics Inc. (Princeton, New Jersey), Trevena Inc. (Chesterbrook, Pennsylvania), awards/grants from the U.S. Food and Drug Administration (Silver Spring, Maryland), and from the Dutch Research Council (The Hague, The Netherlands) for Research Project Tackling and Preventing the Opioid Epidemic (TAPTOE; NWA.1160.18.300). Drs. Pergolizzi and Miller are employees of Enalare Therapeutics Inc. The other authors declare no competing interests.

### Reproducible Science

Full protocol available at: a.dahan@lumc.nl. Raw data available at: a.dahan@lumc.nl.

## Supplemental Digital Content

Supplemental document: Supplemental Table 1 (Pharmacokinetic Parameter Estimates) and Supplemental Figures 1 to 3, https://links.lww.com/ALN/D449

## Supplementary Material
