# Supplementary material for: Reversal of Propofol-induced Depression of the Hypoxic Ventilatory Response by BK-channel Blocker ENA-001: A Randomized Controlled Trial
Source: Anesthesiology. 2024 Jan 19;140(6):1076–87. doi: 10.1097/ALN.0000000000004915 (PMC11097949; doi:10.1097/ALN.0000000000004915)
Supplement: Supplementary file 1 [file aln-140-1076-s001.pdf]

Supplemental materials to:

# Reversal of propofol-induced depression of the hypoxic ventilatory response by BK-channel blocker ENA-001: a randomized controlled trial

Simone C Jansen,[1] Maarten van Lemmen,[1] Erik Olofsen,[1] Laurence Moss,[2] Joseph V Pergolizzi Jr,[3,4] Thomas Miller,[3,5] Robert D Colucci,[4,6] Monique van Velzen,[1] Philip Kremer,[2] Albert Dahan,[1,7] Rutger van der Schrier,[1] Marieke Niesters[1,7]

1. Department of Anesthesiology, Leiden University Medical Center, Leiden, the Netherlands; 2. Centre for Human Drug Research, Leiden, the Netherlands; 3. Enalare Therapeutics Inc., Princeton, New Jersey, USA; 4. NEMA Research Inc., Naples, Florida, USA; 5. Department of Pediatrics, Sidney Kimmel Medical College, Thomas Jefferson University, Philadelphia, Pennsylvania, USA: 6. Colucci & Associates, LLC, Newton, Connecticut, USA; 7. PainLess Foundation, Leiden, the Netherlands

## CONTENTS

Supplemental Table 1. Pharmacokinetic model parameter estimates ………………… Page 2

Supplemental Figure 1. Individual measured plasma ENA-001 concentration. Data fits, and pharmacokinetic goodness-of-fit plots ………………………………………………… Page 3

Supplemental Figure 2. Pharmacodynamic goodness-of-fit plots …………………….. Page 4

Supplemental Figure 3. Population predicted hypoxic/hypercapnic ventilatory responses and influence of propofol and ENA-001 …………………………………………………… Page 5

Supplemental Table 1: Pharmacokinetic parameter estimates.

| Parameter | Estimate ± SEE | Between-subject variability ($\omega^2$) ± SEE | Inter-occasion variability ($\nu^2$) ± SEE |
|---|---|---|---|
| | | **ENA-001** | |
| $V_1$ (L) | 8.3 ± 1.0 | 0.047 ± 0.021 | |
| $V_2$ (L) | 82.4 ± 11.2 | | |
| $CL_1$ (L/h) | 0.88 ± 0.10 | | 0.080± 0.021 |
| $CL_2$ (L/h) | 1.82 ± 0.10 | | 0.067 ± 0.017 |
| $\sigma^2$ | 0.029 ± 0.002 | | |
| | | **Propofol** | |
| V1 (L) | 9.9 ± 2.2 | | 0.388 ± 0.215 |
| V2 (L) | 51.4 ± 5.7 | | |
| CL1 (L/h) | 2.09 ± 0.10 | 0.017 ± 0.008 | 0.024 ± 0.007 |
| CL2 (L/h) | 1.61 ± 0.16 | | 0.035 ± 0.019 |
| $\sigma^2$ | 0.025 ± 0.003 | | |

$V_1$ and $V_2$ are volumes of compartment 1 and 2, $CL_1$ is clearance from compartment 1, $CL_2$ is the intercompartmental clearance. $\sigma^2$ is residual error. SEE is the standard error of the estimate.

Supplemental Figure 1.

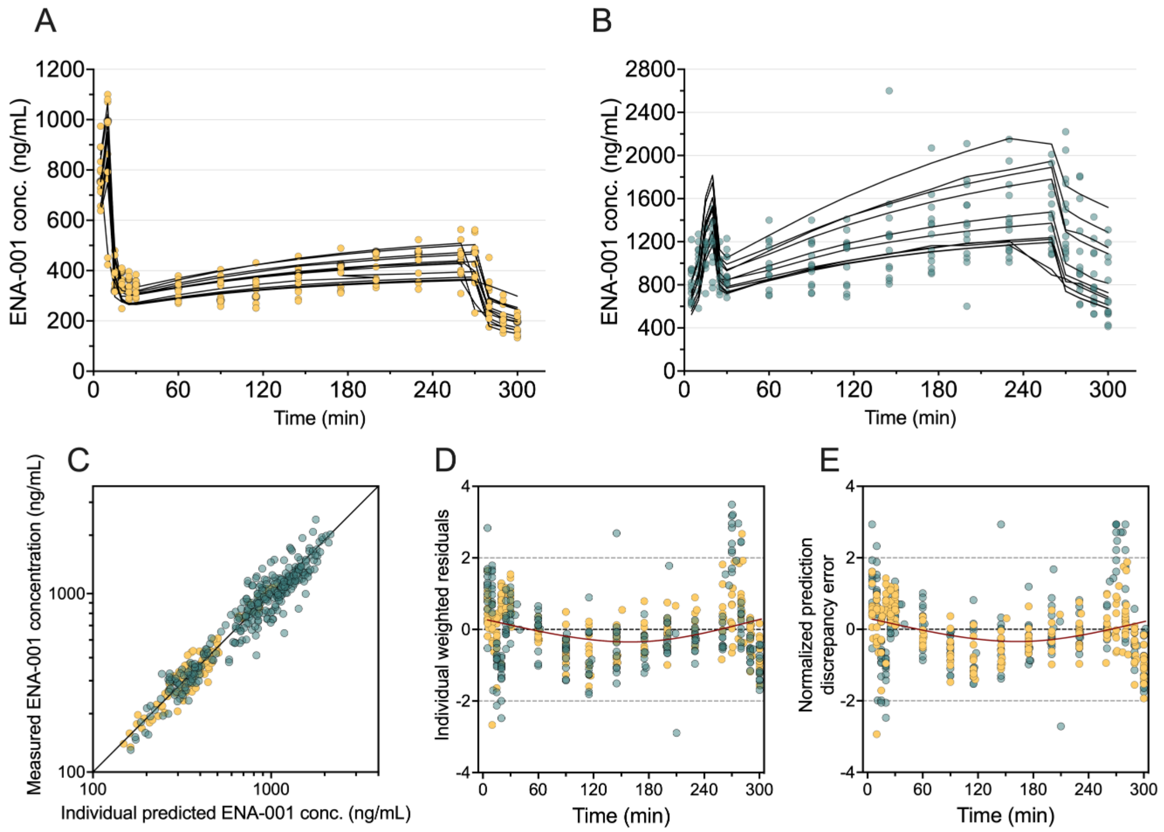


Individuals measured plasma ENA-001 concentrations (yellow and green symbols) following infusion of low-dose (**A**) and high-doe (**B**) ENA-001, with corresponding data fits (continuous lines). **C-D.** Goodness of fit plots of the ENA-001 data fits. **C**. Measured ENA-001 concentration *versus* individual predicted concentrations. **D**. Individual weighted residuals *versus* time. **E.** Normalized prediction discrepancy error *versus* time. The red lines through the data are smoothed curves. Yellow symbols are low-dose ENA-001, green symbols high-dose ENA-001.

Supplemental Figure 2.

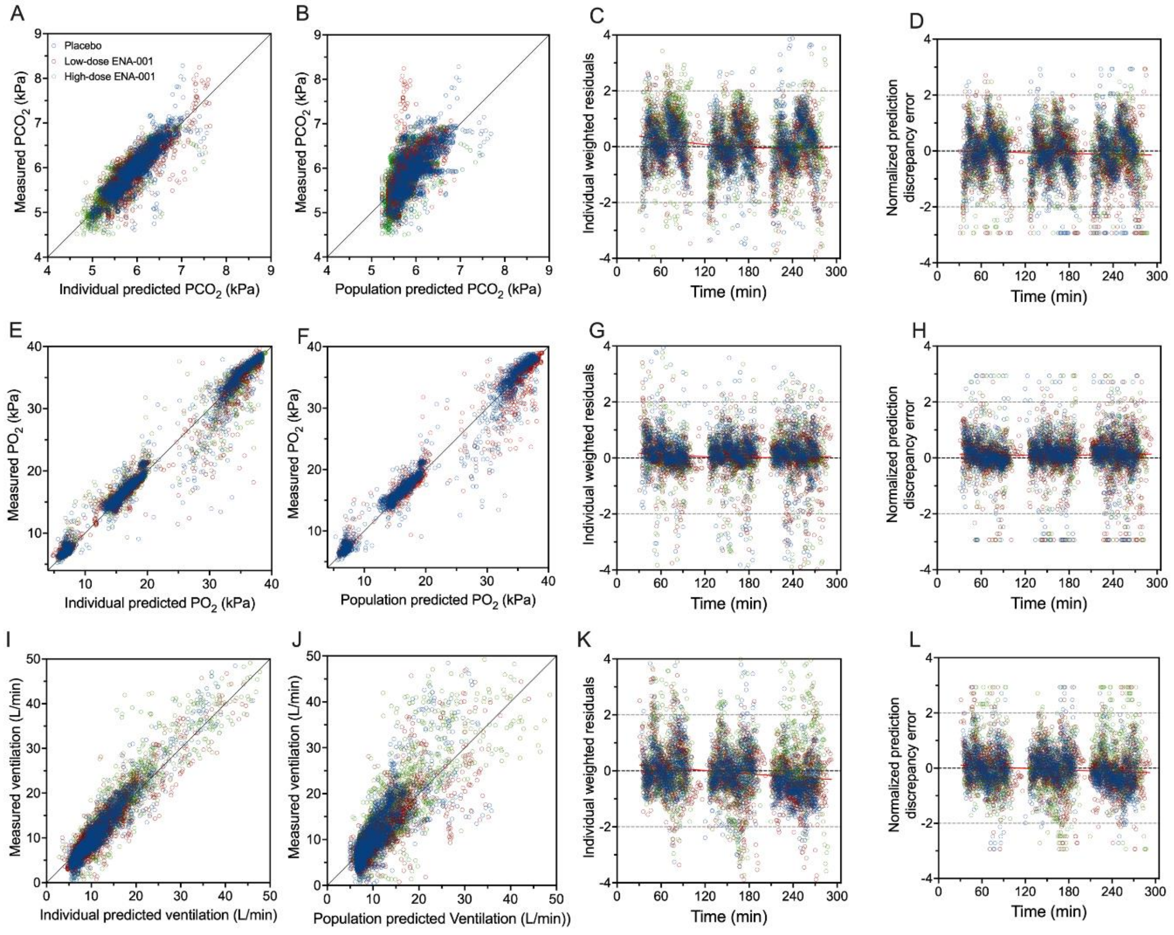


Goodness of fit plots of the population data fits. **A-D.** Alveolar $PCO_2$. **E-H.** Alveolar $PO_2$. **I-L**. Ventilation. **A, E** and **I**. Measured *versus* individual predicted data. **B, F** and **J**. measured *versus* population predicted data. **C, G** and **K**. Individual weighted residuals *versus* time. **D, H** and **L**. Normalized prediction discrepancy error *versus* time. The red lines through the data are smoothed curves. Blue symbols: placebo, red symbols: low-dose ENA-001, and green symbols: high-dose ENA-001.

Supplemental Figure 3.

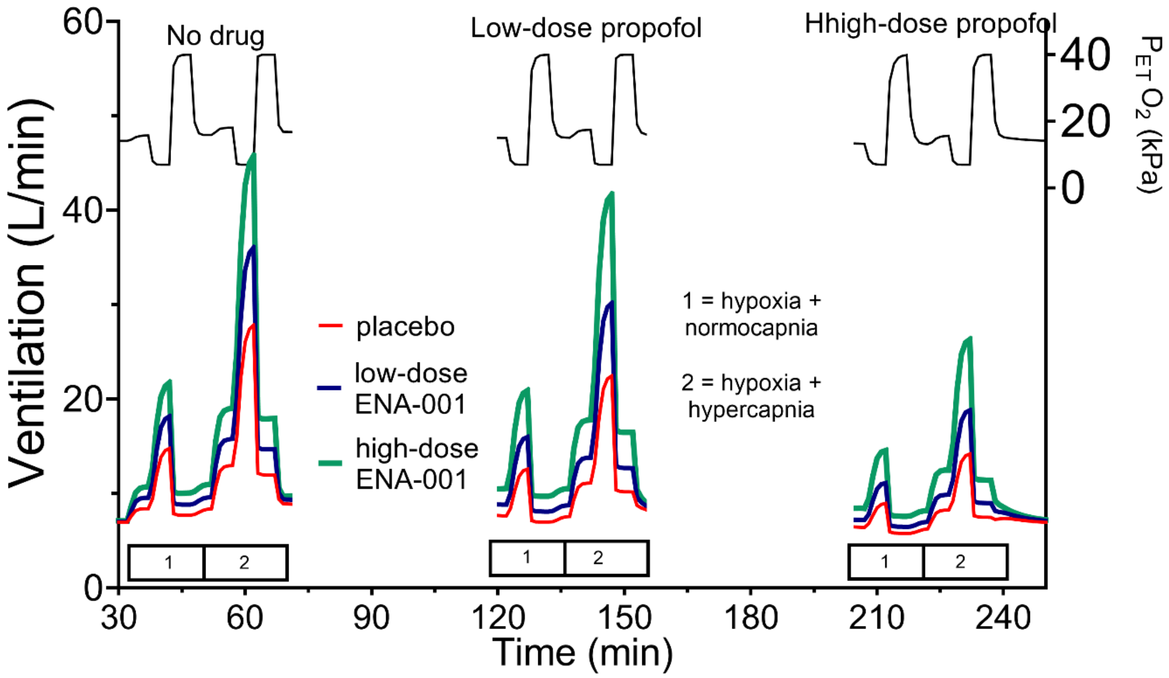


Population predicted ventilatory response to hypoxia + normocapnia (time window 1) and hypoxia + hypercapnia (time window 2) for placebo (red lines), low-dose ENA-001 (blue lines) and high-dose ENA-001 (green lines) during administration of placebo (left), low-dose propofol (middle) and high-dose propofol (right). The top panel depicts the end-tidal oxygen concentration ($P_{ET}O_2$).
